# Clinical Associations with Lenticulostriatal Vasculopathy (LSV) at Birth: A Case–Control Study †

**DOI:** 10.3390/children12020223

**Published:** 2025-02-12

**Authors:** Aikaterini Kyriakopoulou, Kyriakos Samikos, Aikaterini Kanavaki, Efthymia Alexopoulou, Maria Argyropoulou, Theodora Psaltopoulou, Christina Kanaka-Gantenbein, Argyrios Dinopoulos, Melpomene Giorgi, Anastasia Antoniadou, Iliani Filippa, Nikolaos Siafakas, Stylianos Serghiou, Vassiliki Papaevangelou

**Affiliations:** 1 Third Department of Pediatrics, Attikon General University Hospital, National and Kapodistrian University of Athens, 124 62 Athens, Greece; argidino@yahoo.com (A.D.); mel.giorgi@hotmail.com (M.G.); vpapaev@gmail.com (V.P.); 2 Iaso Maternity Hospital, 151 23 Athens, Greece; ksamikos2000@yahoo.gr (K.S.); katerinakan@yahoo.gr (A.K.); iliani.filippa@gmail.com (I.F.); 3 2nd Department of Radiology, General University Hospital Attikon, School of Medicine, National and Kapodistrian University of Athens, 124 62 Athens, Greece; ealex64@hotmail.com; 4 Department of Radiology, Faculty of Medicine, School of Health Sciences, University of Ioannina, 455 00 Ioannina, Greece; margyropou@gmail.com; 5 Department of Hygiene, Epidemiology and Medical Statistics, School of Medicine, National and Kapodistrian University of Athens, 115 27 Athens, Greece; tpsaltop@hotmail.com; 6 Diabetes Center, Division of Endocrinology, Diabetes, and Metabolism, First Department of Pediatrics, Agia Sophia Children’s Hospital, National and Kapodistrian University of Athens, 115 27 Athens, Greece; ckanaka@med.uoa.gr; 7 Fourth Department of Medicine, Attikon General University Hospital, National and Kapodistrian University of Athens, 124 62 Athens, Greece; ananto@med.uoa.gr; 8 Clinical Microbiology Laboratory, Attikon General University Hospital, 124 62 Athens, Greece; nsiaf@med.uoa.gr; 9 Prolaio, Inc., Chicago, IL 60606, USA; stelios.serghiou@gmail.com

**Keywords:** LSV, risk factors, foetal, neonatal, cerebral abnormalities, CMV

## Abstract

Objective: To investigate the clinical characteristics associated with the presence of LSV at birth. Design: Prospective 1:1 case–control study. Setting: Two tertiary neonatal units in Athens, Greece. Patients: Premature neonates (≤36 weeks gestational age) who underwent cerebral ultrasound within the first 3 weeks of life, where LSV was detected. Main outcome measure: Associations between LSV and clinical characteristics at birth. Both unmatched and matched analyses stratifying the study population by gestational week were conducted. Two-sided *p*-values were computed using the likelihood ratio test. Results: This study included 166 participants (83 cases and 83 controls). Neonates with LSV exhibited more concurrent cerebral findings, notably periventricular echogenicity. LSV was correlated with higher z-scores for head circumference and body length. LSV was not associated with congenital CMV. Conclusions: This study indicated a relationship between LSV and increased head circumference and body length. Further research is warranted to explore LSV’s pathophysiological mechanisms.

## 1. Introduction

Typically, the vasculature supplying the deep grey nuclei of the basal ganglia and thalamus is not visible on the cranial ultrasound (cUS) of newborns. However, a foetal insult can trigger an inflammatory vascular response, rendering these vessels echogenic and detectable. Lenticulostriate vasculopathy (LSV) manifests as hyperechogenic vessels in the thalami and basal ganglia of neonates, first described in 1985 by Grant et al. in neonates with cytomegalovirus (CMV) infection [1]. Teele et al. later conducted postmortem histopathological examinations on neonates with similar findings, identifying LSV as a deposition of basophilic material in the arterial walls of the basal ganglia and thalamus [1].

Over the past decade, LSV has been increasingly recognised in neonates with various foetal and neonatal diseases [2]. Numerous studies have documented an association between higher-grade cases of LSV and CMV infection, suggesting its use as a marker of central nervous system (CNS) involvement and sensorineural hearing loss [3]. Recent studies have demonstrated an association between LSV and bronchopulmonary dysplasia of the newborn (BPD), necrotising enterocolitis (NEC), and placental pathology, giving newer insights regarding its role with regard to perinatal insults [4,5]. Despite its growing recognition, the clinical implications of LSV remain unclear. There is no consensus among experts regarding its significance, particularly in infants with congenital CMV infection, where isolated LSV is frequently observed [6].

The inconsistent definitions and variability in conditions of newborns with LSV contribute to uncertainty about its association with pathological perinatal events. Therefore, a more precise definition and standardised diagnostic criteria are crucial to improving diagnostic reliability and understanding of the underlying pathophysiological mechanisms and long-term implications.

This study aims to identify clinical risk factors associated with LSV to better understand the underlying pathophysiological mechanisms involved in its appearance.

## 2. Methods

### 2.1. Selection of Cases and Controls and Data Collection

We conducted a prospective multicentre case–control study in two tertiary hospitals in Athens, Greece (Attikon General Hospital and Iaso Maternity Hospital) from January 2019 to September 2022. We aimed to explore the risk factors associated with LSV by assessing its association with various clinical characteristics in premature neonates. Cases were defined as premature neonates (≤36 weeks gestational age) who underwent cerebral ultrasound within the first 3 weeks of life, where LSV was detected. Each case was matched with a control (absence of LSV) based on the closest gestational age at birth (+/−3 days). Newborns older than 36 weeks were excluded. Given our research group’s interest in congenital CMV (cCMV) and its potential association with LSV, we also tested the neonates for cCMV infection. Since cCMV infection is best diagnosed by identifying the virus in urine or saliva before 3 weeks of age, neonates with LSV detected after this age were excluded. Urine specimens (1–3 mL) were collected by a urine bag and stored at −80 °C to maintain viral DNA integrity. The molecular detection of cytomegalovirus (CMV) in urine samples was performed by Polymerase Chain Reaction (PCR).

The prospective assessment and grading of lenticulostriate vasculopathy (LSV) was performed on the acquired images. Ultrasounds were conducted by paediatric radiologists with at least 10 years of experience in neonatal cerebral imaging. Lenticulostriate vessels were evaluated based on grading systems adapted from recent studies [7]. The neonates were separated into four groups based on the following grading system: A: No LSVs; B: mild (stage 1)—one or two thin branches seen; C: moderate (stage 2)—two to three thin branches seen; D: severe (stage 3)—three prominent thick branches seen [4,8,9].

Even though we gathered numerous characteristics during the study, we defined the following variables as the most relevant and included them in the final statistical analysis: the presence of congenital cytomegalovirus (cCMV) infection, somatometric variables (head circumference, body length, and body weight), sex, intrauterine growth retardation (IUGR), small or large for gestational age (SGA/LGA), presence of any other cerebral findings on cerebral ultrasound (with each abnormal finding independently examined in relation to LSV), maternal gestational diabetes, and gestational hypertension. Furthermore, we note that given that the study included preterm newborns, all somatometric variables were adjusted for gestational age and reported as Z-scores [10]. We adhered to the STROBE checklist to ensure our research met the standards for quality reporting in observational studies.

### 2.2. Ultrasound Assessment

Ultrasound over the anterior and posterior fontanel and asterion (six standard quasi- coronal views and five sagittal views) [11] was performed by an experienced paediatric radiologist using high-frequency transducers (7.5 and 10 MHz LOGIQ V2 and VIVID i by GE Healthcare). No additional neuroimaging was conducted.

### 2.3. Statistical Analysis

Our statistical analysis was adjusted for gestational age in terms of completed full weeks. Even though our study design paired each case to an individual control on the basis of gestational age ± three days, this approach improves statistical power with minimal sacrifice in precision [12].

We computed descriptive statistics using medians and interquartile ranges, which are robust to right and left skew. We quantified differences between cases and controls using the standardised mean difference (SMD). The SMD is commonly used to assess covariate balance between matched cases and controls because respective means may not be directly comparable due to potential influence from the matched variables. It is calculated by taking the difference in means between the cases and controls and dividing it by the mean standard deviation between cases or controls. An SMD < 10% is considered ideal, and an SMD < 20% is considered an acceptable balance [13].

Gestational week-matched differences in numeric variables were tested using the Fisher–Pitman permutation test [14]. Unlike one-way Analysis of Variance (ANOVA), this test does not require the normality assumption [15]. Similarly, matched differences in categorical variables were tested using the Cochran–Mantel–Haenszel test [12]. Unlike McNemar’s test, this allows for more than two strata, each of which can be of any size. Two-sided *p*-values were approximated using the non-parametric bootstrap with 10,000 Monte Carlo resampling with replacement.

Univariable matched odds ratios were estimated using the conditional logistic regression (with exact estimation of the partial likelihood), stratifying by gestational week. Multivariable matched odds ratios were calculated using the same approach, while also adjusting for sex, gestational diabetes, and gestational hypertension. Two-sided *p*-values were computed using the likelihood ratio test.

For completeness, in addition to the matched analysis, we conducted an unmatched analysis utilising standard logistic regression, wherein gestational age was included as a covariate. For both the univariable and multivariable analyses, odds ratios were computed to assess the associations between the variables of interest and the outcome. This analysis can be found in the Supplementary Material (Table S1). All analyses were conducted in R 4.2.2 [16] using the packages coin [17] and survival [18].

## 3. Results

Among the cases, 36 (43.4%) exhibited mild LSV, 41 (49.4%) showed moderate LSV, and 6 (7.2%) displayed severe LSV. The distribution of all variables studied among the three groups of LSV (mild; moderate; severe) is depicted in the Supplementary Material (Table S2).

Between cases and controls, cCMV was detected in two neonates from the control group (2.4%) and in three neonates from the cases group (3.6%, *p* = 0.677) (Table 1). Additionally, other cerebral findings were recorded. Our analysis indicated that neonates with LSV had a significantly higher occurrence of concomitant abnormalities compared to the control group. Specifically, 19 cases (22.9%) and 10 controls (12%) exhibited concomitant abnormalities (*p* = 0.046). Notably, periventricular echogenicity was the most common concomitant finding, observed in 12 cases (14.5%) compared to 5 controls (6.0%) (*p* = 0.038).

We then analysed the birth somatometric measurements and classified them as “abnormal” when any measurement deviated by 2 standard deviations (SD) from the mean for gestational age (GA), corresponding to a z-score of −2 or +2. Based on this assumption, we further examined the association between “abnormal birth somatometric measurements” for GA and the presence of LSV.

Regarding head circumference, the incidence of “abnormal head circumference” was higher in children with LSV (cases) compared to the control group. However, the *p*-value did not reach statistical significance [7 (8.4%) vs. 2 (2.4%), *p* = 0.0873]. A similar pattern was observed for body length, with a greater number of cases exhibiting abnormal measurements compared to controls [6 (7.2%) vs. 3 (3.6%), *p* = 0.492]. Abnormal weight was similar between cases and controls [3 (3.6%) vs. 4 (4.8%), *p* = 0.6983].

When assessing the SMD between the two groups (cases vs. controls), a substantial SMD (greater than 0.2) was observed for multiple variables: z-length (0.403), z-weight (0.283), z-head circumference (0.350), abnormal head circumference (0.268), and other cerebral findings (0.450). In all of these variables, newborns with LSV (cases) exhibited greater weight, length, and head circumference compared to the controls (Table 1).

Initially, a gestational-week-matched univariable analysis was conducted to determine the differences in both numerical and categorical variables between the cases (with LSV) and controls (without LSV). Several variables were found to have a significant association with LSV.

**Figure 1:**
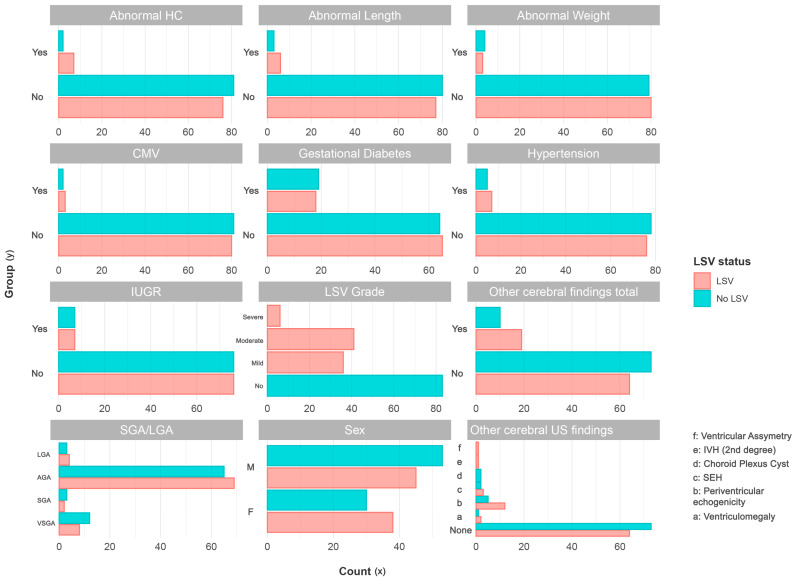
Comparison of categorical variables between neonates with LSV (pink) and neonates without LSV (blue).

Specifically, the variables that demonstrated a significant association with LSV included increased z-length (*p* = 0.011), increased z-head circumference (*p* = 0.019), the presence of other cerebral findings (*p* = 0.044), and the total number of other cerebral findings (*p* = 0.046). These findings indicate that higher z-length, z-head circumference, head circumference, and the presence of other cerebral findings were significantly associated with the occurrence of LSV. A comparison of the categorical variables between neonates with LSV and neonates without LSV showed no significant differences (Figure 1).

### 3.1. Matched Univariable Analysis

**Figure 2:**
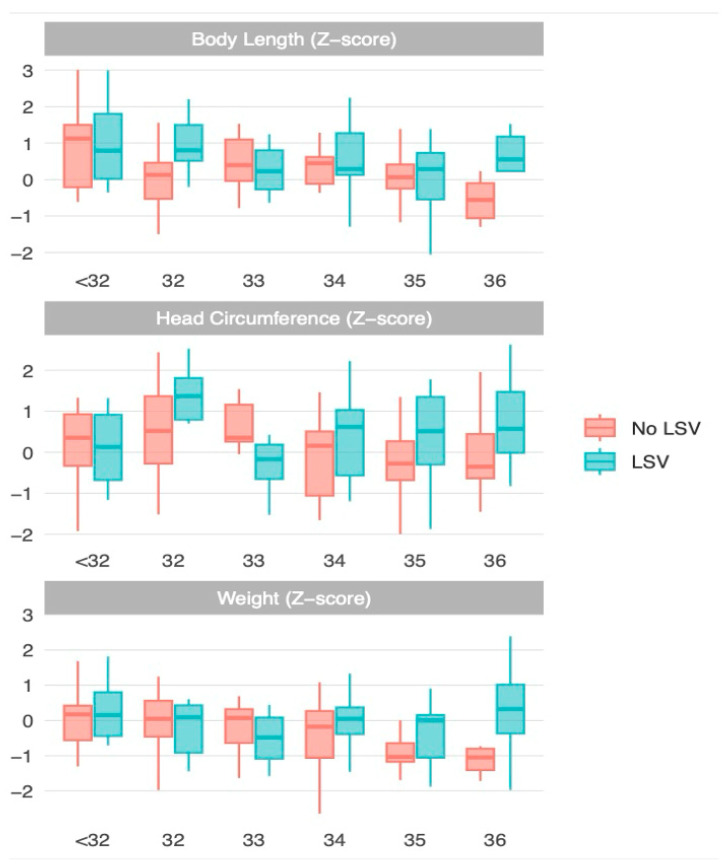
Matched logistic regression analysis demonstrating the distribution of z-length, z-weight, and z-head circumference in neonates with LSV and controls across different gestational ages.

To calculate the univariable matched odds ratios, a conditional logistic regression was performed, grouping the data based on gestational week (Table 2, Figure 2). The presence of cCMV was not associated with the presence of LSV. The variables that showed a significant correlation with LSV were increased z-body length (odds ratio [OR] = 1.51, 95% CI 1.09–2.08, *p* = 0.013), increased z-head circumference (OR = 1.52, 95% CI 1.07–2.16, *p* = 0.020), and the presence of other cerebral findings (OR = 2.80, 95% CI 1.08–7.26, *p* = 0.034). Furthermore, when examining individual cerebral findings separately, LSV was associated with the presence of periventricular echogenicity (OR, 4.39; 95% CI 1.25–15.45; *p* = 0.021).

### 3.2. Matched Multivariable

The multivariable-matched odds ratios were calculated to assess the associations between various variables and LSV (Table 3). The analysis revealed that the same variables remained significantly associated with LSV, even after adjusting for sex, gestational diabetes, and gestational hypertension. Specifically, z-length (OR 1.58, 95% CI: 1.13–2.21, *p* = 0.007), z-head circumference (OR 1.57, 95% CI: 1.10–2.26, *p* = 0.014), and the presence of other cerebral findings (OR 3.09, 95% CI: 1.17–8.15, *p* = 0.022) all showed significant associations with LSV.

### 3.3. Severe LSV vs. Controls

We performed a focused analysis (both matched and unmatched) only on those newborns exhibiting severe LSV (*n* = 6), which did not differ from the aforementioned analysis of the whole LSV group. Congenital CMV infection was not associated with severe (grade 3) LSV. The analysis revealed a significant association between severe LSV and increased z-length (OR: 1.49, 95% CI: 1.04–2.13, *p* = 0.029) as well as with an increased z-head circumference (OR: 1.71, 95% CI: 1.11–2.63, *p* = 0.015) (Supplementary Material Tables S3 and S4).

## 4. Discussion

We performed a prospective 1:1 case–control study matched for gestational age, aiming to explore potential clinical risk factors associated with the presence of LSV on the cerebral US of premature neonates.

Since LSV was initially described in cases of cCMV infection over 30 years ago [19], we were particularly interested in whether the presence of LSV, in conjunction with prematurity—a frequently observed consequence of congenital infections—is associated with congenital CMV infection and could serve as a potential screening biomarker in the future [20].

We found no association between cCMV infection and the presence of LSV. It is, however, important to note that in this small cohort, there were only five children with cCMV (three cases and two in the control group). According to the most recently published guidelines on cCMV, the prognostic implication of isolated lenticulostriate vasculopathy is not yet fully understood, while the decision on the administration of antiviral treatment in infants with isolated LSV is based on expert advice [21,22]. Neonates with LSV had a higher occurrence of concomitant US cerebral findings, and when compared against each separate abnormality, LSV was primarily associated with periventricular echogenicity, potentially implying an underlying component of stress and vascular regulation. Regarding somatometric measurements, an increased z-length and z-head circumference was associated with LSV. These associations remained significant in the multivariable logistic regression, even after adjusting for sex, gestational diabetes, and gestational hypertension. Additionally, a focused analysis of severe LSV cases (n = 6) demonstrated similar results.

Our results from both the matched and unmatched analysis consistently demonstrated the unexpected finding that neonates with LSV exhibited a non-pathological but statistically significant increase in body length and head circumference compared to the controls.

Interestingly, in a recent study, neonates with stage 3 (severe) LSV had significantly higher birth weight when compared to controls and neonates with mild or moderate LSV [4]. However, in this cohort, the authors did not report length or head circumference. Moreover, the same group recently reported that neonates with severe LSV are more likely to have placentas that are large for gestational age (LGA) [5]. Placentomegaly (thickness > 40 mm) has been observed in congenital infections due to placentitis caused by viral transmission through the placenta [23].

To date, various pathophysiological mechanisms have been proposed for LSV. Its association with a vascular pathophysiological mechanism is supported by Doppler studies and histopathological examinations revealing thickened, hypercellular arterial walls with basophilic deposits in the basal ganglia and/or thalamic area [1,24,25]. In the context of congenital infections, LSV has been proposed to represent vasculitis occurring as an inflammatory response to the intrauterine stress caused by the virus. However, it is important to note that since LSV has been described in a large variety of conditions, additional pathophysiological mechanisms have been proposed. Based on histopathophysiological findings such as acute neuronal necrosis and reactive gliosis, mechanisms such as hypoxic–ischaemic injury have also been proposed [26]. This is further supported by the fact that the immature foetal brain has areas of selective vulnerability to insults like hypoxia–ischaemia, with the basal ganglia and thalamus being highly susceptible. The vulnerability of these regions can be attributed to several factors, including the nature of their blood supply from perforating arteries (i.e., lenticulostriatal vessels) with limited capillary anastomosis and autoregulatory responses [27]. Additionally, the basal ganglia and thalamus have high metabolic demands, making them more susceptible to hypoxic–ischaemic injury when autoregulatory responses are disrupted [28]. Repeated episodes of hypoxia–ischaemia and oxidative stress can cause significant damage to these regions, leading to lenticulostriatal vasculopathy (LSV) in the immature brain.

Ultimately, the increase in LSV cases might be linked to recent improvements in Doppler ultrasounds, enabling more precise imaging of intricate vessels. Furthermore, the correlation of LSV with an enlarged head circumference might be due to a technically easier visualisation, potentially amplifying the identification of LSV cases and contributing to the overdiagnosis of a non-pathological finding in neonates with higher HC.

We must acknowledge that this study’s main limitation is the small number of participants; overall, 166 neonates were enrolled (83 with LSV and 83 controls). This resulted in a small number (n = 5) of neonates with cCMV. However, LSV was associated with the presence of periventricular echogenicity, which can certainly imply an underlying component of stress and vascular regulation but may not necessarily be a pathological finding. Additionally, US images were reviewed by a single radiologist, limiting the accuracy of the diagnostic assessments, especially regarding the milder cases of LSV. Finally, the study relied on the initial somatometric measurements and did not include any imaging or neurodevelopmental follow-up data. This limits the ability to capture potential changes over time as well as to aid in elucidating the clinical implications of LSV in child development. Longitudinal assessment would have provided a more comprehensive understanding of the condition and its progression. Furthermore, more confounding variables related to maternal health and prenatal exposures could have been taken into account.

In conclusion, the results of this small case–control study hint at a potential link between severe LSV and larger newborns, suggesting possible compensatory growth responses to stress, which warrants further investigation. This study does not support an association between lenticulostriate vasculopathy (LSV) and congenital CMV. LSV may represent a non-specific cerebral insult. In future studies, improved definitions and identification methods for LSV in neonatal cranial ultrasounds are essential for enhancing diagnostic accuracy and understanding its implications for neonatal outcomes.

## Figures and Tables

**Table 1:** Descriptive characteristics and group comparisons of neonates with LSV (cases) and neonates without LSV (controls).

Characteristic	Controls	Cases	SMD	*p*-Value
	N = 83	N = 83		
CMV (%)	2 (2.4)	3 (3.6)	0.071	0.677
Male sex (%)	53 (63.9)	45 (54.2)	0.197	0.280
Gestational hypertension (%)	5 (6.0)	7 (8.4)	0.093	0.553
Gestational diabetes (%)	19 (22.9)	18 (21.7)	0.029	1.000
Head circumference				
z-head (mean (SD))	0.24 (0.92)	0.57 (0.96)	0.350	0.019
abnormal head circumference (%)	2 (2.4)	7 (8.4)	0.268	0.087
Weight				
z-weight (mean (SD))	−0.38 (0.97)	−0.11 (0.92)	0.283	0.083
abnormal weight (%)	4 (4.8)	3 (3.6)	0.060	0.698
Body length				
z-length (mean (SD))	0.04 (1.01)	0.45 (1.01)	0.403	0.011
abnormal length (%)	3 (3.6)	6 (7.2)	0.160	
IUGR (%)	7 (8.4)	7 (8.4)	<0.001	1.000
Birth weight for gestational age (%)			0.175	0.800
VSGA (<3rd percentile)	12 (14.5)	8 (9.6)		
SGA (<10th percentile)	3 (3.6)	2 (2.4)		
AGA (10th <> 90th percentile)	65 (78.3)	69 (83.1)		
LGA (>90th percentile)	3 (3.6)	4 (4.8)		
LSV grade (%)				
n.a.	83 (100.0)	0 (0.0)		
mild	0 (0.0)	36 (43.4)		
moderate	0 (0.0)	41 (49.4)		
severe	0 (0.0)	6 (7.2)		
Other cerebral findings total (%)	10 (12.0)	19 (22.9)	0.289	0.044
Other cerebral findings (%)			0.450	0.046
no other findings	73 (88.0)	64 (77.1)		
ventriculomegaly	1 (1.2)	2 (2.4)		
periventricular echogenicity	5 (6.0)	12 (14.5)		
SEH bilaterally	2 (2.4)	3 (3.6)		
choroid plexus cyst	2 (2.4)	0 (0.0)		
second-degree IVH	0 (0.0)	1 (1.2)		
ventricular asymmetry	0 (0.0)	1 (1.2)		

**Table 2:** Conditional logistic regression analysis of variables in neonates with lenticulostriate vasculopathy (LSV).

Matched Univariable Conditional Logistic Regression						
Predictor	Odds Ratio (OR)	SE	Z-Score	*p*-Value	CI (Low)	CI (High)
z-length	1.51	0.16	2.492	0.013	1.09	2.08
z-head	1.52	0.18	2.317	0.02	1.07	2.16
Periventricular echogenicity	4.39	0.64	2.305	0.021	1.25	15.45
Other cerebral findings total	2.80	0.49	2.12	0.034	1.08	7.26
z-weight	1.35	0.17	1.718	0.086	0.96	1.89
Abnormal head circumference	4.06	0.82	1.711	0.087	0.82	20.22
Sex	0.70	0.31	−1.161	0.246	0.38	1.28
SHE bilaterally	2.83	1.09	0.954	0.34	0.33	23.85
Abnormal length	1.97	0.73	0.921	0.357	0.47	8.29
Ventriculomegaly	2.31	1.23	0.681	0.496	0.21	25.69
LGA	1.79	0.91	0.64	0.522	0.3	10.62
Hypertension	1.48	0.61	0.639	0.523	0.45	4.89
Abnormal weight	0.63	0.8	−0.571	0.568	0.13	3.03
CMV	1.58	0.92	0.499	0.618	0.26	9.61
IUGR	1.10	0.56	0.179	0.858	0.37	3.29
Gestational diabetes	0.94	0.38	−0.15	0.881	0.44	2.01
SGA	0.96	1.03	−0.037	0.97	0.13	7.3
Choroid plexus cyst	0.00	6319.37	−0.003	0.998	0	Inf
Second-degree IVH	284,000,000	8944.37	0.002	0.998	0	Inf

**Table 3:** Multivariable logistic regression analysis of variables in neonates with lenticulostriate vasculopathy (LSV).

Matched Multivariable Regression (Adjusted by Sex, Gestational Diabetes, and Hypertension)						
Predictor	Odds Ratio (OR)	SE	Z-Score	*p*-Value	CI (Low)	CI (High)
z-length	1.58	0.17	2.7	0.007	1.13	2.21
z-head	1.57	0.18	2.457	0.014	1.1	2.26
Periventricular echogenicity	4.86	0.65	2.423	0.015	1.35	17.48
Other cerebral findings total	3.09	0.49	2.285	0.022	1.17	8.15
z-weight	1.41	0.18	1.898	0.058	0.99	2.01
Abnormal head circumference	4.07	0.83	1.7	0.089	0.81	20.56
SEH bilaterally	2.97	1.08	1.009	0.313	0.36	24.61
Abnormal length	1.91	0.74	0.871	0.384	0.45	8.15
LGA	2.05	0.94	0.765	0.444	0.33	12.8
Abnormal weight	0.58	0.81	−0.67	0.503	0.12	2.85
Ventriculomegaly	2.14	1.23	0.616	0.538	0.19	23.9
CMV	1.49	0.93	0.428	0.669	0.24	9.22
IUGR	1.10	0.57	0.167	0.867	0.36	3.35
SGA	0.97	1.05	−0.028	0.977	0.12	7.67
Choroid plexus cyst	0.00	6337.58	−0.003	0.998	0	Inf
Second-degree IVH	220,000,000	8968.6	0.002	0.998	0	Inf

## Data Availability

The data presented in this study are available on request from the corresponding author. The data are not publicly available due to the anonymous nature of the study.

## Supplementary Materials

The following supporting information can be downloaded at https://www.mdpi.com/article/10.3390/children12020223/s1. Supplementary Table S1. Univariable & Multivariable Unconditional Logistic Regression Analysis of Variables in Neonates with Lenticulostriate Vasculopathy (LSV). Supplementary Table S2. Distribution of Characteristics among Subgroups of Neonates with Lenticulostriate Vasculopathy (LSV): Mild, Moderate, and Severe Cases. Supplementary Table S3. Unconditional Logistic Regression Analysis of Variables in Neonates with Severe Lenticulostriate Vasculopathy (LSV). Supplementary Table S4. Conditional Logistic Regression Analysis of Variables in Neonates with Severe Lenticulostriate Vasculopathy (LSV).
