# Supplementary material for: Clinical Associations with Lenticulostriatal Vasculopathy (LSV) at Birth: A Case–Control Study
Source: Children (Basel). 2025 Feb 12;12(2):223. doi: 10.3390/children12020223 (PMC11853899; doi:10.3390/children12020223)
Supplement: Supplementary file 1 [file children-12-00223-s001.zip › children-3397474-supplementary.pdf]

Appendix

Unmatched Logistic Standard Regression (univariable & multivariable analysis)

In addition to the matched analysis, we conducted both univariable and multivariable unmatched analysis by adjusting for gestational age in a logistic regression (Table 4)[14]. We aimed to assess the association between predictor variables and the presence of LSV when taking into account gestational age. The results of this analysis confirmed the findings obtained in the matched analysis, regarding the variables that demonstrated significant associations with LSV among the cases.

When taking gestational age into account, results revealed that increased body length (OR = 1.49, 95% CI: 1.10 - 2.06, p = 0.012), increased z-weight (OR = 2.45, 95% CI: 1.07 - 6.04, p = 0.041), increased head circumference (OR = 1.28, 95% CI: p = 0.028), increased z-head circumference (OR = 1.51, 95% CI: 1.07 - 2.16, p = 0.012), and the presence of other cerebral abnormalities in total (OR = 2.62, 95% CI: 1.07 - 2.16, p = 0.012) were all positively associated with LSV. Supplamentery Table 1 demonstrates the results of the univariable and multivariable unmatched analysis.

	**Univariable**			**Multivariable** Adjust by sex, gestational diabetes, gestational hypertension		
**Predictors**	**Odds Ratios (OR)**	**P-values**	**95 % CI**	**Odds Ratios (OR)**	**P-values**	**95 % CI**
**z-length**	1.49	0.012	1.1-2.06	1.58	0.006	1.15-2.2
**z-head**	1.51	0.021	1.07-2.16	1.58	0.013	1.11-2.29
**Periventricular echogenicity**	3.78	0.031	1.19-13.9	4.11	0.024	1.27-15.5
**Other cerebral findings total**	2.62	0.043	1.06-6.95	2.80	0.032	1.12-7.51
**z-weight**	1.37	0.065	0.99-1.95	1.45	0.041	1.02-2.09
**Abnormal head circumference**	3.92	0.099	0.9-27.3	3.94	0.099	0.89-27.4
**Sex**	0.67	0.209	0.36-1.25	n/a	n/a	n/a
**SEH Bilaterally**	3.05	0.277	0.41-27.9	2.99	0.285	0.41-27.1
**Abnormal length**	2.07	0.315	0.53-10.1	2.02	0.337	0.5-9.99
**LGA**	2.07	0.417	0.36-13.2	2.47	0.325	0.41-16.5
**Ventriculomegaly**	2.26	0.512	0.21-49.4	2.05	0.565	0.19-45.4
**Hypertension**	1.44	0.556	0.43-5.1	n/a	n/a	n/a
**CMV**	1.53	0.649	0.25-11.8	1.41	0.715	0.22-11.1
**Abnormal weight**	0.73	0.688	0.14-3.45	0.68	0.625	0.12-3.28
**Gestational diabetes**	0.92	0.834	0.44-1.95	n/a	n/a	n/a
**SGA**	0.96	0.969	0.11-7.22	0.99	0.991	0.11-7.61
**IUGR**	1.00	1	0.33-3.05	1.00	0.993	0.32-3.1

Supplementary Table 1. Univariable & Multivariable Unconditional Logistic Regression Analysis of Variables in Neonates with Lenticulostriate Vasculopathy (LSV)

Characteristic	Cases	LSV Grade		
		Mild	Moderate	Severe
	N=83	N=36	N=41	N=6
**cmv (%)**	3 (3.6)	1 (2.8)	2 (4.9)	0 (0.0)
**male sex (%)**	45 (54.2)	16 (44.4)	26 (63.4)	3 (50.0)
**gestational hypertension (%)**	7 (8.4)	5 (13.9)	2 (4.9)	0 (0.0)
**gestational diabetes (%)**	18 (21.7)	7 (19.4)	10 (24.4)	1 (16.7)
**head circumference**				
z-head (mean (SD))	0.57 (0.96)	0.45 (0.97)	0.71 (1.00)	0.26 (0.41)
abnormal head circumference (%)	7 (8.4)	2 (5.6)	5 (12.2)	0 (0.0)
**weight**				
z-weight (mean (SD))	-0.11 (0.92)	-0.40 (1.01)	0.16 (0.82)	-0.26 (0.43)
abnormal weight (%)	3 (3.6)	1 (2.8)	2 (4.9)	0 (0.0)
**body length**				
z-length (mean (SD))	0.45 (1.01)	0.16 (1.07)	0.77 (0.92)	-0.03 (0.54)
abnormal length (%)	6 (7.2)	1 (2.8)	5 (12.2)	0 (0.0)
**IUGR (%)**	7 (8.4)	5 (13.9)	2 (4.9)	0 (0.0)
**birth weight for gestational age (%)**				
VSGA (<3rd percentile)	8 (9.6)	7 (19.4)	1 (2.4)	0 (0.0)
SGA (<10th percentile)	2 (2.4)	1 (2.8)	1 (2.4)	0 (0.0)
AGA (10th <> 90th percentile)	69 (83.1)	28 (77.8)	35 (85.4)	6 (100.0)
LGA (>90th percentile)	4 (4.8)	0 (0.0)	4 (9.8)	0 (0.0)
**other cerebral findings total (%)**	19 (22.9)	6 (16.7)	9 (22.0)	4 (66.7)
**other cerebral findings (%)**				
no other findings	64 (77.1)	30 (83.3)	32 (78.0)	2 (33.3)
ventriculomegaly	2 (2.4)	2 (5.6)	0 (0.0)	0 (0.0)
periventricular echogenicity	12 (14.5)	4 (11.1)	6 (14.6)	2 (33.3)
SEH Bilaterally	3 (3.6)	0 (0.0)	1 (2.4)	2 (33.3)
choroid Plexus Cyst	0 (0.0)	0 (0.0)	0 (0.0)	0 (0.0)
second degree IVH	1 (1.2)	0 (0.0)	1 (2.4)	0 (0.0)
ventricular Asymmetry	1 (1.2)	0 (0.0)	1 (2.4)	0 (0.0)

Supplementary Table 2 Distribution of Characteristics among Subgroups of Neonates with Lenticulostriate Vasculopathy (LSV): Mild, Moderate, and Severe Cases.

We performed a focused analysis (both matched and unmatched) only on the severe LSV cases which showed a significant association with increased z-length (OR 2.17, 95% CI 1.44 - 3.47, P = 0.00), z-weight (OR 1.98, 95% 1.28 - 3.23, P = 0.003) and z-head circumference (OR 1.86, 95% CI 1.26 - 2.96, P = 0.007). Furthermore, severe LSV was significantly associated with large for gestational age (LGA) neonates (OR 24.80, 95% CI 2.3 - 670, P = 0.018) as well as those with abnormal head circumference (OR 6.30, 95% CI 1.23 - 47.5, P = 0.0038).

These consistent findings from both the matched and unmatched univariable analyses indicate that neonates with greater z-body length, z-weight, and z-head circumference are more likely to exhibit LSV as observed on cerebral ultrasound, when compared to the control group.

**Severe Unmatched Logistic Regression**						
**Predictors**	**Odds Ratios (OR)**	**SE**	**Z-scores**	**P-values**	**CI (low)**	**CI (high)**
**z-length**	2.17	0.22	3.483	0	1.44	3.47
**z-weight**	1.98	0.23	2.921	0.003	1.28	3.23
**z-head**	1.86	0.23	2.721	0.007	1.21	2.96
**LGA**	24.80	1.36	2.364	0.018	2.3	670.00
**Abnormal head circumference**	6.30	0.89	2.073	0.038	1.23	47.50
**Periventricular echogenicity**	3.69	0.7	1.868	0.062	0.95	15.60
**Other cerebral findings total**	2.66	0.56	1.749	0.08	0.89	8.18
**Abnormal length**	3.59	0.76	1.686	0.092	0.83	18.30
**SEH Bilaterally**	3.77	1.5	0.882	0.378	0.13	109.00
**SGA**	3.67	1.56	0.833	0.405	0.12	115.00
**IUGR**	0.54	0.83	-0.735	0.462	0.08	2.39
**CMV**	2.07	1.02	0.712	0.477	0.24	17.90
**Hypertension**	0.76	0.87	-0.324	0.746	0.1	3.73
**Sex**	0.92	0.4	-0.195	0.845	0.42	2.06
**Gestational diabetes**	1.03	0.46	0.068	0.946	0.41	2.49
**Abnormal weight**	0.95	0.9	-0.055	0.956	0.13	5.21
**Choroid Plexus Cyst**	0.00	1691.47	-0.009	0.993		2.30E+108
**Second degree IVH**	93,400,000	2399.54	0.008	0.994	0	
**Ventricular Asymmetry**	33,200,000	2399.54	0.007	0.994	0	
**Ventriculomegaly**	0.00	2399.54	-0.006	0.995		3.88E+205

Supplementary Table 3. Unconditional Logistic Regression Analysis of Variables in Neonates with Severe Lenticulostriate Vasculopathy (LSV)

Severe Matched Logistic Regression						
**Predictors**	**Odds Ratios (OR)**	**SE**	**Z-scores**	**P-values**	**CI (low)**	**CI (high)**
**z-head**	1.71	0.22	2.439	0.015	1.11	2.63
**z-length**	1.49	0.18	2.179	0.029	1.04	2.13
**Other cerebral findings total**	2.48	0.54	1.673	0.094	0.86	7.16
**Periventricular echogenicity**	3.07	0.71	1.583	0.113	0.77	12.32
**z-weight**	1.35	0.19	1.56	0.119	0.93	1.98
**Abnormal head circumference**	2.96	0.82	1.325	0.185	0.59	14.79
**CMV**	4.41	1.19	1.249	0.212	0.43	45.3
**Gestational diabetes**	0.56	0.47	-1.216	0.224	0.22	1.42
**SEH Bilaterally**	3.85	1.29	1.043	0.297	0.31	48.62
**LGA**	3.41	1.26	0.977	0.329	0.29	40.03
**Abnormal weight**	0.47	0.85	-0.893	0.372	0.09	2.47
**Hypertension**	1.64	0.63	0.781	0.435	0.47	5.68
**Sex**	0.81	0.35	-0.604	0.546	0.41	1.6
**Abnormal length**	1.60	0.77	0.604	0.546	0.35	7.28
**IUGR**	1.43	0.65	0.551	0.581	0.4	5.08
**SGA**	0.65	1.04	-0.412	0.681	0.09	4.99
**Ventriculomegaly**	211,000,000	10733.1	0.002	0.999	0	Inf
**Choroid Plexus Cyst**	0.00	10955.23	-0.002	0.999	0	Inf

Supplementary Table 4. Conditional Logistic Regression Analysis of Variables in Neonates with Severe Lenticulostriate Vasculopathy (LSV)
